# Acute management of fractures in primary care - a cost minimisation analysis

**DOI:** 10.1186/s12913-023-10297-x

**Published:** 2023-11-23

**Authors:** Stein Vabo, Egil Kjerstad, Steinar Hunskaar, Knut Steen, Christina Brudvik, Tone Morken

**Affiliations:** 1 National Centre for Emergency Primary Health Care, NORCE Norwegian Research Centre, Box 22, Nygårdstangen, Bergen, NO-5838 Norway; 2 Vennesla Health Care Centre, Sentrumsvegen 41, Vennesla, NO-4700 Norway; 3 Department of Social Science and Health, Health Services and Health Economics Research Group, NORCE Norwegian Research Centre, Nygårdsgaten 112, Bergen, NO-5008 Norway; 4 Department of Global Public Health and Primary Care, University of Bergen, P.O. Box 7800, Bergen, NO-5020 Norway; 5 Minor Injury Department, Orthopaedic Division, Haukeland University Hospital, Bergen, Norway; 6 Department of Clinical Medicine, University of Bergen, P.O. Box 7800, Bergen, NO-5020 Norway

**Keywords:** Economics, Evaluation, Fractures, Conservative treatment, Family practice, Primary healthcare, Teleradiology, Diagnosis

## Abstract

**Background:**

In Norway, primary healthcare has first-line responsibility for all medical emergencies, including traumas and fractures. Normally, patients with suspected fractures are referred to specialist care in hospitals. However, the cooperating municipalities of Bykle and Valle have X-ray facilities and handle minor fractures locally. The aim of this study was to estimate the costs of X-ray diagnosis and initial treatment of fractures at the local primary care centre compared with initial transport and treatment in hospital.

**Methods:**

We conducted a cost minimisation analysis by comparing expected costs of initial examination with X-ray and treatment of patients with fractures or suspected fractures at two possible sites, in the local municipality or at the hospital. A cost minimisation analysis is an economic evaluation based on the assumption that the outcomes of the two treatment procedure regimens are equal. Costs were estimated in Euros (EUR) using 2021 mean exchange rates.

**Results:**

In 2019, we identified a total of 403 patients with suspected fractures in the two municipalities. Among these, 12 patients bypassed the primary care system as they needed urgent hospital care. A total of 391 injured patients were assessed with X-ray at the primary health care centres, 382 received their initial treatment there, and nine were referred to hospital. In an alternative hospital model, without X-ray and treatment possibilities in the municipality, the 382 patients would have been sent directly to hospital for radiological imaging and treatment. The total cost was estimated at EUR 367,756 in the hospital model and at EUR 69,835 in the primary care model, a cost saving of EUR 297,921.

**Conclusion:**

Based on cost minimisation analysis, this study found that radiological diagnosis of suspected fractures and initial treatment of uncomplicated fractures in primary care cost substantially less than transport to and treatment in hospital.

## Background

Over the last decades, the increase in healthcare costs in developed countries has shown a need for stricter prioritising and more cost-effective use of available resources. A main cost-containing principle in the healthcare sector is to treat patients at the lowest effective level of care [1]. In Norway, this has been a guiding principle in public and political debate and planning for many years. In 2012, the Coordination Reform was implemented, entailing the transfer of medical treatment from the specialist health service level to the primary healthcare service level [2]. In addition, a new regulation in 2015 described how the management of acute injuries outside hospitals should be organised [3]. This regulatory instruction set specific requirements for competence for all health personnel on duty as well as equipment and vehicles.

In many fields of primary care, treatment at this level is considered to be of comparable quality to the specialist level. This applies to the treatment of several diseases and therapies, previously treated in hospital, but now commonly treated in general practice [2]. Acute treatment and short-term observation in the primary healthcare service have also previously been shown to be performed to the same quality as in hospitals, and with lower costs [4].

In Norway, health care is organized in a two - tiered system. All patients are initially evaluated at a primary care level and treated at this level if possible, and only if medically needed are they referred to hospitals and specialists for diagnosis and treatment. Usually, patients with possible fractures are evaluated at primary care level and referred hospitals for X-ray diagnosis and further treatment at this level. However, some municipalities in Norway and elsewhere in Europe have invested in X-ray facilities and treat fractures in primary healthcare [5, 6].

In a previous study from a primary healthcare centre at a ski resort, we have shown that less than a fifth of patients diagnosed with fractures in their wrist, collarbone or ankle were sent to hospital for operative treatment of the fractures [6]. Most patients were initially treated at the primary care level. Follow-up control of the fracture treatment was in most cases carried out at the hospitals’ outpatient clinics after 7–10 days, while some were followed up at the primary healthcare centre. Another study from the same municipality showed low rates of functional disability and high rates of satisfaction after initial diagnosis and treatment of these fractures [7].

Some studies have shown the financial benefits of teleradiology, i.e., performing X-ray examinations in the primary health service and transferring images for reading at the hospital radiological department [8, 9]. Several studies have compared conservative and operative treatment of fractures in hospital [10–12].

In some of the studies, cost analysis has been carried out to compare conservative treatment with plaster, bandages and operative treatment. The studies conclude that conservative fracture treatment has lower costs than operative treatment [13–16].

However, research of economic issues concerning X-ray examinations combined with acute fracture treatment at primary care level, versus treatment at the nearest hospital, is scarce.

It seems highly plausible that there is a substantial cost saving by doing x - ray diagnosis and initial fracture treatment at primary care compared to hospital level. We wanted to quantify this cost saving.

The aim of this study was to estimate the costs of X-ray diagnosis and initial treatment of fractures at the local primary care centre compared with initial transport for treatment in hospital.

## Methods

### Setting

The emergency care system in Norway has two levels: primary healthcare in the municipality and secondary healthcare in hospital. Emergency primary care services are provided by general practitioners during office hours and at emergency primary care out-of-hours services. The hospital system is responsible for the ambulance services. In an emergency, patients are usually first assessed by a primary care physician. After assessment, the patient is either treated locally in primary care or sent to hospital for diagnostic work-up and treatment. In some severe emergencies, for instance involving multi-traumas, patients are transported directly to hospital, usually after telephonic contact with the physician at the primary healthcare centre, but otherwise bypassing the primary care system.

Bykle and Valle are two small municipalities containing popular ski destinations. The two municipalities cooperate on primary healthcare services and were merged into one healthcare unit in 2013. The ski destinations are located in the southern part of Norway, with up to 20,000 ski tourists present daily during peak season, such as Easter and winter holidays. Most of the injuries that cause fractures occur on the alpine skiing slope [6]. Local treatment of fractures is motivated by a long distance to hospital (156 to 210 km), with a transport time of up to three hours by car or ambulance.

### Design of the study

We conducted a cost minimisation analysis to compare the expected cost of treating patients in a primary care model and in an alternative hospital model, respectively. Cost minimisation analysis is an economic evaluation based on the assumption that the patient outcome of the two treatment procedure regimens is equal, despite the different competence (equipment and specialisation), diagnostic and therapeutic opportunities in the two settings [17]. In this cost minimisation analysis, the costs of treatment at the local primary healthcare centre were compared with treatment in hospital [4, 8, 9]. In the primary care model, with X-ray facilities in the municipality and with the necessary trained healthcare personnel, most patients with uncomplicated fractures are treated primarily at the local healthcare centre. In the alternative hospital model, all patients with suspected fractures are sent to hospital for assessment and treatment.

### Primary care model in Bykle and Valle

In Bykle and Valle municipalities, an injured patient is transported to the local healthcare centre for further examination, and the physician on duty decides whether there is indication for X-ray imaging. X-rays are taken by a nurse or physician, and the images are read by the physician. After the clinical assessment and X-ray imaging the physician decides whether the patient should be transferred to hospital for further treatment or treated locally at the primary healthcare centre. If in doubt, the physician discusses the case with the hospital-based orthopaedic surgeon. For quality purposes the primary care physician’s reading of the X-ray images is sent to the radiologist at the hospital for a final reading there. This reading is transferred back and included in the patient’s medical record at the healthcare centre.

In a typical consultation with a suspicion of fracture after an injury, the assessment by the primary care physician, including the X-ray examination, lasts for 30 min or less. If no fracture is found on the X-ray, the patient is sent home with relevant treatment, such as painkillers or bandages.

If the X-ray reveals a fracture, the primary care physician treats the fracture according to the guidelines, sometimes after consultation with the radiologist or orthopaedic surgeon at the hospital. Management of a fracture without need of repositioning will have a consultation time of up to 50 min. If repositioning is necessary, the consultation time is usually 50–65 min.

### Hospital model

In the “normal model” in Norway, patients with injuries are primarily assessed in primary care, and if a fracture is suspected, the patient is transported to hospital for an X-ray and further treatment. The primary care physician calls the hospital, completes X-ray referral papers and provides the receiving hospital with relevant medical information. Transport is then requested electronically or by telephone. A few patients with severe fractures, misalignment, severe pain, or unclear conditions, are sent directly to hospital by ambulance, taxi or helicopter. The primary care physician assesses some patients at the injury site and some through contact with the ambulance personnel by phone or healthcare radio connection.

### Sample and data collection

We included all patients registered with fractures or suspected fractures in the municipalities of Bykle and Valle in 2019. The following years 2020 and 2021 were not appropriate due to the Covid-19 pandemic, which dramatically affected activities at the ski resort. However, we used 2021 prices for the various cost elements.

To investigate the number of X-ray examinations of clinically suspected and X-ray verified fractures at the primary healthcare centre we used the finance part of the medical records. When an electronic medical record is written for an individual patient, the different clinical activities of the primary care doctor generate specific reimbursement codes to HELFO (Norwegian Health Economics Administration). By searching for this code in the medical record system’s economic module, we found the number of X-rays performed. Treatment of a fracture with no need for repositioning is another code, while a third code gives the number of fractures with a need for repositioning and control X-ray after treatment [18].

By searching the medical record system for fractures coded with the International Classification of Primary Care-2 (ICPC-2) diagnostic codes L72, L73, L74, L75, L76, L83, L84 and L95, we found the number of fractures [19]. All records with these diagnostic codes were read by the author (SV) and we identified whether the fracture was confirmed by a radiologist, and whether the patient received primary treatment locally at the primary care centre or was sent directly to hospital by ambulance after a local X-ray examination.

We registered transport from the two different primary healthcare centres (Bykle and Valle) to the hospital. Patients sent directly to hospital by ambulance, due to fractures or injuries with an urgent need for diagnosis and treatment in hospital, were not included in the cost minimisation analysis, as the cost would be identical in both the primary care model and the hospital model.

### Estimating costs

#### Consultation and treatment

Table 1 shows the elements of estimated costs in the primary care model and in the hospital model, respectively.

**Table 1:** Estimated costs of elements for treatment of patients with suspected fractures in primary care and hospital (2021) (see Fig. 1)

		Primary care costs (EUR)			Hospital costs (EUR)	
Cost type		Day	Night		Day	Night
Consultation (20–35 min)		50	62		199	212
Consultation (35–50 min)		72	81		199	212
Consultation (50–105 min)		94	99		199	212
X-ray imaging		33	33		28	28
Simple cast		27	27		115	115
Repositioning and cast		52	52		176	176
Taxi transport Valle 156 km round trip and waiting time*		0	0		525	642
Taxi Bykle 210 km round trip and waiting time*		0	0		660	818
Treatment group, cost model (combined costs, transportation excluded)						
	A	83	95		227	239
	B	132	141		314	327
	C	179	184		375	387

*Taxi transport cost is calculated by using the following prices: Cost Day: EUR 2.5 per km, Cost night: EUR 3.25 per km. Waiting time: From arrival at hospital to return, an estimated three hours of waiting, EUR45 per hour

To estimate the costs of the primary care model, we used the tariffs from the collective agreement between the Norwegian Medical Association and the Ministry of Health and Care Services [11]. In primary care, the cost is dependent on the medical procedures performed, the duration of the consultation, the time of day, material costs and medical expenses. (casts, elastic bandages, painkillers, local anaesthetic medicines). For the hospital model, costs are calculated on the basis of the relevant codes from the Diagnosis Related Groups (DRG) based remuneration system, the associated DRG weights and the price per DRG point in 2021 [20]. The cost in EUR (weight*EUR per DRG point) reflects estimations based on data collected from a sample of Norwegian hospitals, i.e., medical procedures, use of bandage material and the time spent on consultations and care, etc., and can be interpreted as the average cost across Norwegian hospitals for a given procedure or treatment. We did not attempt to allocate the share of basic funding of the hospital service to our cost estimation, i.e., basic funding is treated as a fixed cost irrespective of the number of patients admitted from Bykle and Valle. However, we added costs in primary care for consultation, contact with the hospital, referral papers and organising transport, prior to the hospital attendance.

Obviously, the cost of conservative fracture treatment in primary care and hospital depends on the injury sustained and the treatment given for each patient. We divided the patients into five groups (Table 2): (A) No fracture on X-ray and treatment with elastic bandages or painkillers only; (B) Confirmed fracture on X-ray, but no need for repositioning, treatment with cast; (C) Confirmed fracture on X-ray, need for repositioning, treatment with cast; (D) Confirmed fracture on X-ray, referral to hospital without further treatment in primary care; and (E) Clinical fracture with or without other complicating traumas, sent directly to hospital by ambulance without consultation in primary care and prehospital X-ray.

**Table 2:** Treatment groups managed in primary care or in the alternative hospital model (2019)

Group	Clinical features	No. of patients	Content of management in primary care, including X-ray	Alternative hospital-based management, without X-ray in primary care
**A**	No fracture on X-ray and no further treatment.	273	Consultation, including X-ray (20–35 min)	Consultation in primary care (20–35 min), contact hospital, referral notes for X-ray and outpatient clinic, order transport. In hospital: Consultation, including assessment of X-ray by physician at orthopaedic outpatient clinic.
**B**	Confirmed fracture on X-ray, but no need for repositioning, treatment with cast.	80	Consultation, including X-ray and treatment with cast (35–50 min)	Consultation in primary care (20–35 min), contact hospital, referral notes for X-ray and outpatient clinic, order transport. In hospital: Consultation, including assessment of X-ray by physician at orthopaedic outpatient clinic, treatment with cast.
**C**	Confirmed fracture on X-ray, need for repositioning, treatment with cast.	29	Consultation, including X-ray and X-ray control after cast (50–65 min)	Consultation in primary care (20–35 min), contact hospital, referral notes for X-ray and outpatient clinic, order transport. In hospital: Consultation, including assessment of X-rays by physician at orthopaedic outpatient clinic, repositioning and treatment with cast.
**D**	Confirmed fracture on X-ray, referral to hospital without further treatment in primary care.	9	Consultation, including X-ray (20–35 min)	Consultation in primary care (20–35 min), contact hospital, referral notes for X-ray and outpatient clinic, order transport. In hospital: Consultation, including assessment of X-rays by physician at orthopaedic outpatient clinic, treatment with cast, surgery or other.
**E**	Clinical fracture with or without other complicating traumas, sent directly to hospital by ambulance without consultation in primary care.	12	No consultation, with or without communication with ambulance paramedics.	No consultation in primary care. In hospital: Consultation, including assessment of X-rays by physician at orthopaedic outpatient clinic, treatment with cast, surgery or other.

The cost of consultation and treatment in primary care depends on time of consultation and/or treatment, i.e., day, night, or weekend. Based on our experience in the municipality, we assumed that 50% of the patients are treated and transported during the daytime, and 50% at night/during the weekend. The activity is high at weekends, and the fractures during the day are often treated in the late afternoon and evening. The cost of treatment in hospital is identical regardless of day or time of admission.

#### Transportation to hospital

In our calculation, we assumed one hospital transport per injury and one patient per transportation to hospital. The public patient travel system in Norway compensates the patient’s transportation expenses to and from publicly approved medical treatment venues. To reduce costs, they prefer to coordinate and include more patients in the same vehicle for each transport. However, this practice is not applicable to patients with acute fractures who need of urgent hospital care.

In 2021, the base price for taxi transport was EUR 2.5 per km for long-distance transport during the daytime [21]. The transport distance to hospital is 156 km (25% of the patients) from the nearest healthcare centre and 210 km from the most distant healthcare centre (75% of the patients).

In the hospital model, all patients with suspected fractures are transported to hospital by taxi or ambulance for X-ray examination and treatment at an orthopaedic outpatient clinic, or admission for surgery [14]. Most of the patients do not need surgery, and in these cases the taxi will wait for the patient, to make the return journey. The waiting time was set at three hours on average, estimated by local taxi drivers. The price per hour is EUR 45.

### X-ray machines

In 2019, there was one X-ray machine in each of the two municipalities. The cost of the two machines, acquired in 2014 and 2016 respectively, totalled EUR 168,400. The service life per machine is at least ten years. The cost per year is set at 10% of initial value, i.e., EUR 16,840, and in addition service and upgrade per year of EUR 12,460, a total of EUR 29,300 per year.

The hospital cost of X-ray machines was set at 0, as we assumed that the cost of X-ray machines at a hospital would be similar in both the primary care model and hospital model.

### Other costs

In the primary care model, personnel will need a short introductory course to perform X-rays, but no more than usual when other types of new medical equipment are introduced. The X-ray machines have automatic settings, and it is easy to obtain high quality pictures. The learning process needed to perform X-rays-images is short. This training can be considered part of the regular updates that healthcare professionals must receive. Due to a low radiation risk from ordinary X-ray imaging, the current X-ray equipment can be used without lead protection and in an ordinary room that is otherwise used for other medical activities. Based on these considerations, we did not estimate any costs of training or extra office rooms in the primary care model.

## Results

**Fig. 1:**
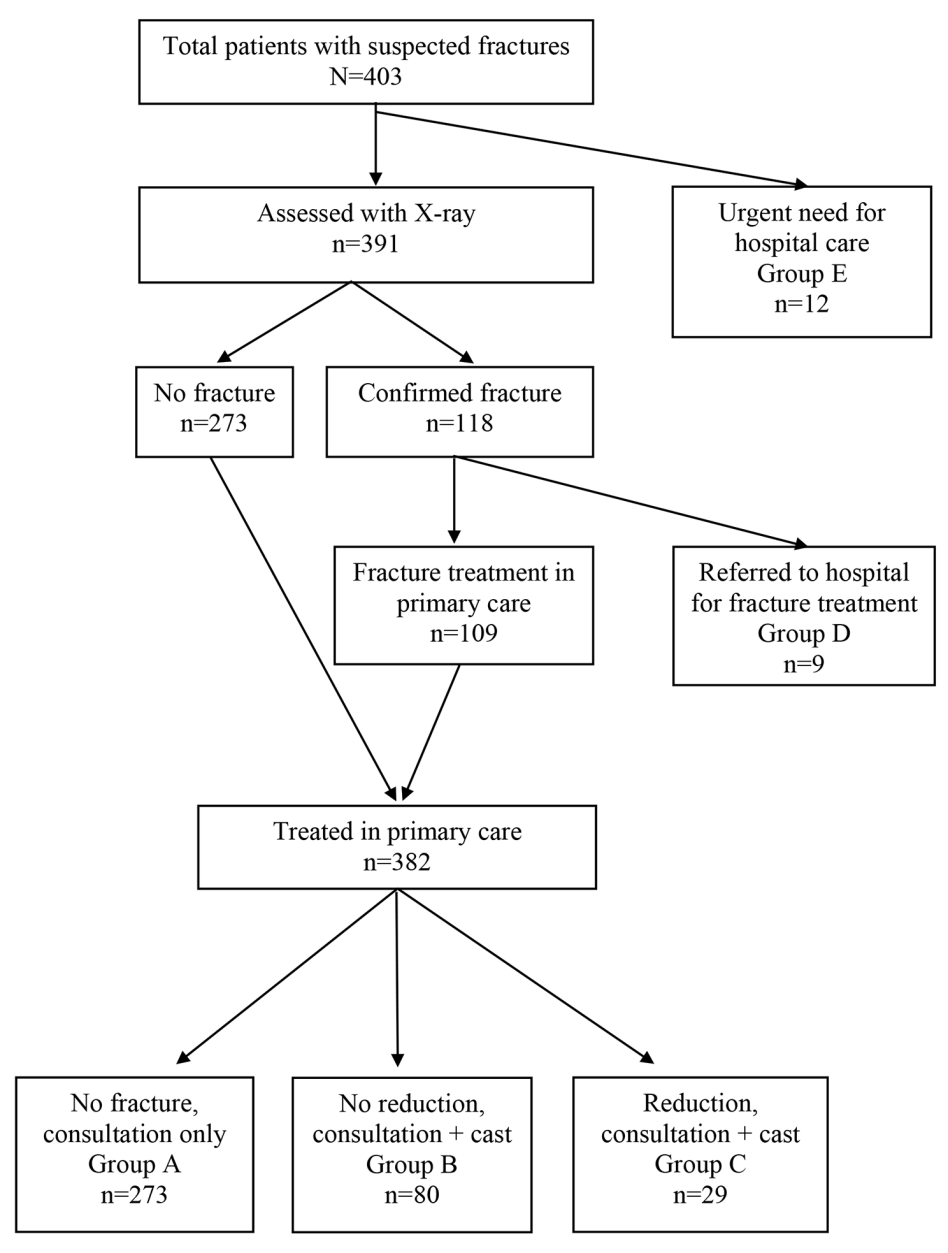
Flow chart for patients with suspected fractures

In 2019, we identified a total of 403 patients with suspected fractures in Bykle and Valle (Fig. 1). Among these, 12 patients bypassed the primary care system due to a need for urgent hospital care. In total 391 patients were examined with X-rays at the primary healthcare centre and included in the study. A total of 382 patients were treated locally at the primary healthcare centre, and 118 (30%) of these patients had fractures confirmed by X-ray. Nine of the 118 patients with fractures were referred to hospital for fracture treatment and 109 patients were treated at the primary healthcare centre. Of the patients treated in primary care, 80 patients were treated with casts and 29 patients were treated with repositioning and casts. We found that 75% of the primary treatment procedures for fractures took place in Bykle and 25% in Valle municipality.

The cost of treatment for the 382 locally treated patients was estimated at EUR 40,535 in the primary care model and EUR 100,338 in the alternative hospital model, i.e., EUR 59,803 more in the hospital model (Table 3). The total taxi transport cost, which is only generated in the hospital model, was estimated at EUR 267,418 based on the tariffs for 2021. The annual cost of operating X-ray equipment in the municipalities was estimated at EUR 29,300. The total cost of initial transport and treatment of the 382 patients in the hospital model was estimated at EUR 297,921 more than for X-ray diagnosis and initial treatment of the patients in the primary care model.

**Table 3:** Estimated costs for 382 patients in different treatment groups in the primary care model and the hospital model (see Fig. 1)

	Primary care model (EUR)			Hospital model (EUR)	
Treatment cost					
	Average cost*	Total cost		Average cost*	Total cost
A (n = 273)	89	24,297		233	63,609
B (n = 80)	137	10,960		321	25,680
C (n = 29)	182	5,278		381	11,049
Total treatment cost		40,535			100,338
**Transport cost**					
A, B, C (n = 382)					
Valle (n = 96)	0	0		584	56,064
Bykle (n = 286)	0	0		739	211,354
Total transport cost					267,418
**X-ray cost 2021**	29,300	29,300		0	0
**Total cost**		69,835			367,756

*Average cost is calculated assuming 50% of patients as daytime work and 50% as night work

We estimated number of patients to cover the annual cost of the X-ray service in our municipalities.

In our study, fracture was diagnosed for 30% of the patients; 2% were sent to hospital; 28% were treated at the primary healthcare centre; 21% were treated with casts and bandages without repositioning; and 8% after repositioning. 70% had no fracture detected at the X-ray examination. If this distribution is used as a basis, we must treat 187 patients in the primary care model to cover the annual costs of the X-ray service at the primary care centre in our municipalities.

## Discussion

In this study we calculated the difference in costs between two models for acute management of fractures: the primary care model and the hospital model. We found significant cost savings when X-ray diagnosis of suspected fractures, as well as initial treatment of uncomplicated fractures, were performed in primary care, compared to transportation of patients to hospital for X-rays and treatment.

### Transport

The largest cost-driving element in the hospital model was the transportation costs, which accounted for almost two thirds of the total cost. The transportation costs are large but will naturally depend on the distance from the location of injury to the nearest hospital. In the primary care model, the transport of patients is avoided. In our study, it turned out that for 70% of the X-ray examinations made, no fracture was detected. This saved these patients from unnecessary hospital visits and long and time-consuming transportation. Similarly, a Dutch study of teleradiology found that approximately 60% of the X-ray examinations showed no fracture, and patients avoided five hours of unnecessary transport to the nearest hospital [5].

Our transport cost estimate is based on the assumption that all non-ambulance transport is by means of taxi. But some of these patients will use private cars, which will trigger a lower cost in the hospital model.

### Treatment

The treatment cost in the hospital model was significantly higher, more than double, compared to the primary care model. Firstly, the actual cost when treated in the hospital is higher. Secondly, the hospital model will have a cost triggered from the primary healthcare service for assessing and referring the patient. When treating in primary care rather than referring the patient, there will be no hospital cost. The principle of treating at the lowest possible management level, and referring, when necessary, implies a good management of the gatekeeper role to primary physician as well, namely only referring to hospital injuries that needs to be treated in the hospital. In this setting it reduces the cost substantially.

Nevertheless, even if the primary care step was bypassed, the primary care model would still be less expensive than the hospital model. If the financial reimbursement for fracture treatment and X-rays in primary care was doubled, the primary care model would still be cheaper.

Few studies have compared the costs of treatment in primary healthcare versus treatment in hospital, and none dealing with fracture treatment. However, a study by Vibeto et al. [4] compared treating patients with acute poisoning by substances of abuse in a primary care emergency clinic with treatment in hospital, which is the usual strategy. The estimated cost per patient in primary care was EUR 121, and EUR 612 in the hospital model. This is approximately a fifth of the cost is the hospital model. In our study, the estimated average cost per patient in primary care was EUR 106, and treatment in the hospital model was estimated at EUR 267, showing a two and a half times higher cost in the hospital model.

### X-ray

In the primary care model, X-ray equipment at the primary healthcare centre is a necessary extra investment for treating fractures. However, the X-ray cost was relatively low, even though the two municipalities needed one machine each. As the cost of X-ray equipment has diminished in recent years, the extra cost of equipment of satisfactory quality in primary care will be of even less importance in a cost minimisation analysis. Several other studies have shown cost savings when using X-rays in primary care for diagnostics of trauma as well as non-traumatic conditions [8, 9].

If we solely consider the costs of the X-ray examinations and treatment in the primary care model in our study and compare them with the hospital model, it is of interest that we only need to treat 187 patients before the primary care model becomes cost-saving. However, if X-ray examination and fracture treatment were implemented in a primary care centre or municipality in 2021, the estimated cost could be even lower e.g., EUR 50,000 for X-ray- equipment, including service for ten years, i.e., an annual cost of EUR 5,000. Then only 32 patients with suspected fractures in a year would be needed to cover the annual X-ray cost to break even with the cost of the hospital model.

### Other costs

We did not include the cost of ambulances in the study, as the increase in expected ambulance transfers was assumed to be relatively small. However, transport of all patients in the hospital model may result in a need for extra ambulances and even higher costs during periods of high activity at the ski destination. Another consequence of more ambulance transfers is that the ambulance with its personnel will be out of the municipality for long periods, which will weaken the emergency response in the municipality during those periods.

Costs to the patients or next of kin are not included in our cost minimisation analysis. Adult patients may suffer a loss of income due to the time spent on transport for examination and treatment in hospital. When children and young people are injured, parents or other care givers must take time off work to accompany the patient to and from the hospital where the treatment takes place. The productivity loss for society caused by long transport times and waiting times for next of kin is likely to be smaller in the primary care model compared to the hospital model. Avoiding transportation also means avoiding longer periods of pain and discomfort for patients.

### Generalisability

As Norway is a country with long distances, we think that the cost saving potential from transferring treatment of uncomplicated fractures from hospitals to primary care centres is significant for some municipalities with high incidence of fractures. Most ski destinations in Norway that are remote from hospital are already equipped with digital X-ray equipment and conduct fracture treatment like the municipalities in our study. However, we think that some other rural primary care centres could undertake the same acute management of fractures, with significant cost savings for society.

This is probably the case in other countries as well. Here too, local treatment of fractures in the primary health care due to a long transport distance to hospital, will have the greatest potential for being cost saving.

### Strengths and limitations

A strength of this study is that we have relatively complete data concerning what we wanted to investigate. The extracts from the medical record system covered almost 100% of the X-ray examinations, transportation and treatments. We also carefully sought information about the different cost factors associated with these elements.

A weakness is that some of the other cost elements are difficult to quantify, so that some assumptions must be made in relation to these elements. In our economic estimates, we have chosen to disregard these. An example is increased use of ambulances.

The costs calculated in this study are estimated solely from the health care system perspective. A broader societal perspective would include many other factors, e.g., costs to the patient or next of kin for loss of income and transport time but is beyond the scope of this study. Another limitation in this study is that we were not able to stratify cost by age/gender or high/low energy traumas. It was not possible to extract these figures from our database. 

## Conclusion

Based on this cost minimisation analysis, the economic evaluation based on the assumption that the outcomes of the two treatment procedure regimens are equivalent, we found that X-ray diagnosis and initial treatment of fractures at the local primary care centre in a high-risk area for injury and fracture cost substantially less than transport to and treatment in hospital. Likewise, if all transportation costs were removed, the primary care model would still be cheaper.

### Implications and further research

A main cost-containing principle in the healthcare sector is to treat patients at the lowest effective level of care. Our study supports the assumption that teleradiology makes it possible to treat conditions previously treated in hospital at a significantly lower cost in primary care [4]. This knowledge should impact the discussion about cost-effective use of available resources, in the choice between primary care and care at the hospital level. Despite lower costs for implementation of X-ray equipment, there is still a need for more research of the cost savings from fracture treatment in primary care in other municipalities and other patient populations.

## Data Availability

The datasets used and/or analysed during the current study are available from the corresponding author on reasonable request.

## Declarations

### Author’s information

The first author of this article (SV) has been a specialist in general medicine since 1996. In the 2005–2011 period he was chief physician and general practitioner in Bykle municipality and responsible for the quality of the medical work performed in the municipality.
